# A Quantitative Documentation of the Composition of Two Powdered Herbal Formulations (Antimalarial and Haematinic) Using Ethnomedicinal Information from Ogbomoso, Nigeria

**DOI:** 10.1155/2014/751291

**Published:** 2014-02-20

**Authors:** Adepoju Tunde Joseph Ogunkunle, Tosin Mathew Oyelakin, Abosede Oluwaseyi Enitan, Funmilayo Elizabeth Oyewole

**Affiliations:** Environmental Biology Unit, Department of Pure and Applied Biology, Ladoke Akintola University of Technology, PMB 4000, Ogbomoso, Oyo State, Nigeria

## Abstract

The safety of many African traditional herbal remedies is doubtful due to lack of standardization. This study therefore attempted to standardize two polyherbal formulations from Ogbomoso, Oyo State, Nigeria, with respect to the relative proportions (weight-for-weight) of their botanical constituents. Information supplied by 41 local herbal practitioners was statistically screened for consistency and then used to quantify the composition of antimalarial (Maloff-HB) and haematinic (Haematol-B) powdered herbal formulations with nine and ten herbs, respectively. Maloff-HB contained the stem bark of *Enantia chlorantha* Oliv. (30.0), *Alstonia boonei* De Wild (20.0), *Mangifera indica* L. (10.0), *Okoubaka aubrevillei* Phelleg & Nomand (8.0), *Pterocarpus osun* Craib (4.0), root bark of *Calliandra haematocephala* Hassk (10.0), *Sarcocephalus latifolius* (J. E. Smith) E. A. Bruce (8.0), *Parquetina nigrescens* (Afz.) Bullock (6.0), and the vines of *Cassytha filiformis* L. (4.0), while Haematol-B was composed of the leaf sheath of *Sorghum bicolor* Moench (30.0), fruit calyx of *Hibiscus sabdariffa* L. (20.0), stem bark of *Theobroma cacao* L. (10.0), *Khaya senegalensis* (Desr.) A. Juss (5.5), *Mangifera indica* (5.5), root of *Aristolochia ringens* Vahl. (7.0), root bark of *Sarcocephalus latifolius* (5.5), *Uvaria chamae* P. Beauv. (5.5), *Zanthoxylum zanthoxyloides* (Lam.) Zepern & Timler (5.5), and seed of *Garcinia kola* Heckel (5.5). In pursuance of their general acceptability, the two herbal formulations are recommended for their pharmaceutical, phytochemical, and microbial qualities.

## 1. Introduction

The Medical and Dental Practitioners (Amendment) Decree number 78 promulgated by the federal government of Nigeria on September 30, 1992, placed traditional and alternative medicine side by side with the orthodox medicine [1]. Ever since that time, there had been an overwhelming increase in the public awareness and usage of herbal medicines in the treatment and/or prevention of diseases. This development has been attributed to the active mass media advertisement embarked upon by the manufacturers and marketers of herbal drugs who have taken advantage of the relatively high cost of the conventional medicines [2].

In an attempt to enhance the acceptability of herbal medicines by consumers in Nigeria, some manufacturers, licensed by the National Agency for Food Drug Administration and Control (NAFDAC), and, also, many unregistered local practitioners have come up with products usable in the conventional dosage forms such as tablets, capsules, suspensions, solutions, and powders [2]. Many of these herbal products usually contain two or more botanicals each of which has a number of chemical compounds that may give the anticipated activity in combination.

In the development of polyherbal formulations, pharmacognosists undertake to analyze and evaluate those active ingredients from different medicinal plants for their possible chemical interactions with various excipients [3, 4]. Information on which such studies are based usually originates from the traditional healers who make use of the herbs without any form of standardization. Annexure I of the guidelines published by the World Health Organization on evaluation of traditional medicine provides, among others, for quantitative list of active ingredients to accompany each herbal product for the information of the consumer. In cases where the active ingredients have not been identified, the whole herb serving as a constituent of a multiherbal formulation is regarded as one active ingredient [5]. At the best, traditional herbal remedies display their constituent plant species and sometimes, also, the plant parts with little or no empirical information on their relative proportions [6, 7]. Such details, especially to the traditional African herbalists, constitute a trade secret that must be jealously guarded. This practice has effectively hindered the development of phytomedicine in this part of the world.

Although traditional herbal medicine has always been part of the people's culture in Africa, this form of medicine is yet to be relatively well organized as, for example, in India and China [8]. African herbal products have particularly been called to question on account of adulteration, substitution, contamination, misidentification, lack of standardization, incorrect preparation and/or dosage, inappropriate labeling, and/or advertisement [9, 10]. Also in the view of Idika and Niemogha [11], there is an urgent need for developing some systems and methods of standardization for traditional medicines in order to enhance the general acceptability, to allay the skepticism and fears of the people, to avoid dangers of toxicity, side effects, and overdosage, and to ensure that a certain minimum level of hygiene in their preparation is maintained.

Malaria fever has been listed as a major public health problem in Nigeria where it accounts for more cases and deaths than any other country in the world [12, 13]. It is responsible for over 70% of outpatient hospital visitation and has a great toll on productivity, being a major cause of absenteeism from school and work and of disease and complications in children and pregnant women [14]. There is also evidence that people in Nigeria now attend hospitals as often as they go to herbalists to treat themselves of this dreaded disease, which is most prevalent in southwestern, north central, and northwestern parts of the country [12, 15, 16].

Much as in the orthodox medical practice, herbalism in many parts of the world, including southwestern Nigeria, considers the care of the blood as paramount to preventive and curative health care [7, 17, 18]. Against this background, the present study sought to address the issue of standardization of antimalarial and haematinic powdered herbal remedies popularly used in Ogbomoso, Nigeria, by way of documenting their prevalent botanical composition, parts used, and relative proportions of these “active ingredients.”

## 2. Materials and Methods

### 2.1. Preliminary Survey and Data Manipulation

A preliminary survey among 55 local herbal practitioners (HPs) in Ogbomoso land, Nigeria, in May 2011 revealed that 41 of them were noble in the production and sale of both types of herbal remedies of interest. The questionnaire-guided survey made the respondents identify the botanicals by name and parts used for the antimalarial herbal remedy (AMHR) and haematinic herbal remedy (HAHR).

Before the choice of a statistical tool for analyzing the data collected, it was found expedient to accept that the statistical mode of each of the two distributions among the 41 HPs should be taken as the most typical herbal formulation in the study area. To that extent, qualitative data on the botanical composition of both remedies were independently transformed into frequency distribution tables, each with class interval size of 2. With the observation of the maximum frequency occurring at the end of the two distributions, the method of grouping three classes at a time was adopted [19] and the mode in each case was calculated using the formula according to Gupta [19] as follows:

(1) $$\begin{array}{c} \text{Mode}=l+\frac{h\left|f_{1}-f_{0}\right|}{\left|f_{1}-f_{0}\right|+\left|f_{1}-f_{2}\right|}, \end{array}$$

where *l* is lower limit of the modal class, *f*_1_ is frequency of the modal class, *f*_0_ is frequency of the class preceding the modal class, *f*_2_ is frequency of the class succeeding the modal class, and |*A*| is the absolute (positive) value of *A*.

The exercise described above, which, respectively, yielded 9 and 10 herbs as modes for AMHR and HAHR successfully reduced the number of HPs to be included to 32, all of which listed both* Enantia chlorantha *Oliv. and *Alstonia boonei* De Wild as part of AMHR and *Sorghum bicolor* Moench. as part of HAHR. For consistency in the list of the plants in each remedy, three further respondents were excluded, such that the number of HPs to be included in the next phase of the study was 29. Considering the fact that data for the two herbal remedies under study were independent and that some healers offered one while others offered both remedies, it was possible to identify 33 herbal product outlets (HPOs) from among the 29 HPs, comprised of 19 for AMHRs and 14 for HAHRs.

### 2.2. Determination of Percent Composition of Herbal Fractions

Between December 2011 and April 2012, visits were made to the 33 identified HPOs to seek information concerning the source of plant materials used, relative quantity of each fraction, and procedure for preparing the powdered AMHRs and HAHRs. An open plastic container of approximately 5-litre volume (20 cm top diameter, 17 cm bottom diameter, and 18 cm depth) was used as a standard measure, which each respondent was asked to reference for the final powdered product. The respondents were made to select the quantity of each dried herbal component as if they were to carry out the exercise on a good day. The selections were procured into labeled plastic bags with the assumption that the corresponding herbs collected from different outlets had been dried to fairly equal moisture contents.

The weights (g) of the nine components of AMHR procured from 19 outlets and the ten components of HAHR from 14 outlets were each determined using a Measuretech triple-beam balance MB-2610 model. In recognition of the necessity for data consistency in this type of study, the weight values for each remedy were fed as multivariate data matrix into the computer-based SPSS version 17.0, which was used to identify and exclude outliers across the treatments (i.e., the outlets) in each case. This step reduced the number of outlets for final consideration to 25, that is, 14 and 11 for AMHR and HAHR, respectively (Table 1).

Using the same statistical software, two other analyses were carried out on the quantitative data for the two remedies. These were Pearson's correlation test on the treatments and the means of the variables (i.e., herbal fractions). The total of the mean values of the herbal components in a remedy was equated to 100% and the contribution of each component was computed with reference to 100.

## 3. Results and Discussion

**Figure 1:**
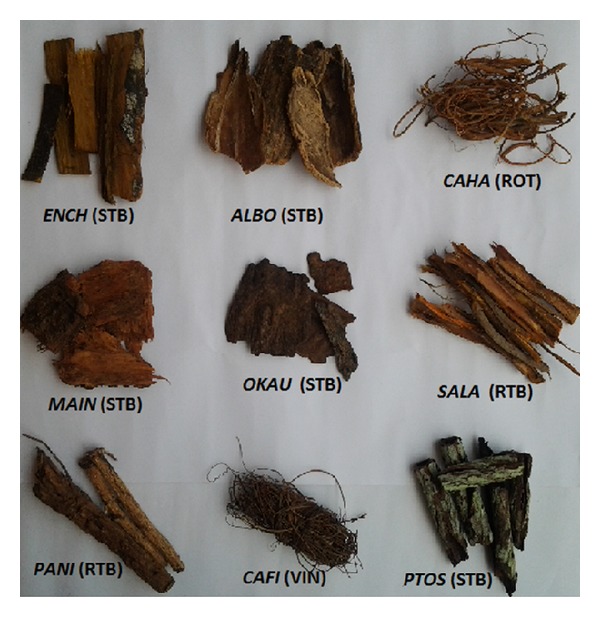
Samples of the herbs used in combination as antimalarial herbal formulation (Maloff-HB) in Ogbomoso, Nigeria. *ENCH: Enantia chlorantha*; *ALBO: Alstonia boonei*; *CAHA: Calliandra haematocephala*; *MAIN: Mangifera indica*; *OKAU*: *Okoubaka aubrevillei*; *SALA*: *Sarcocephalus latifolius*; *PANI*:* Parquetina nigrescens*; *CAFI*: *Cassytha filiformis*; *PTOS: Pterocarpus osun*; STB: stem bark; ROT: root; VIN: vines.

**Figure 2:**
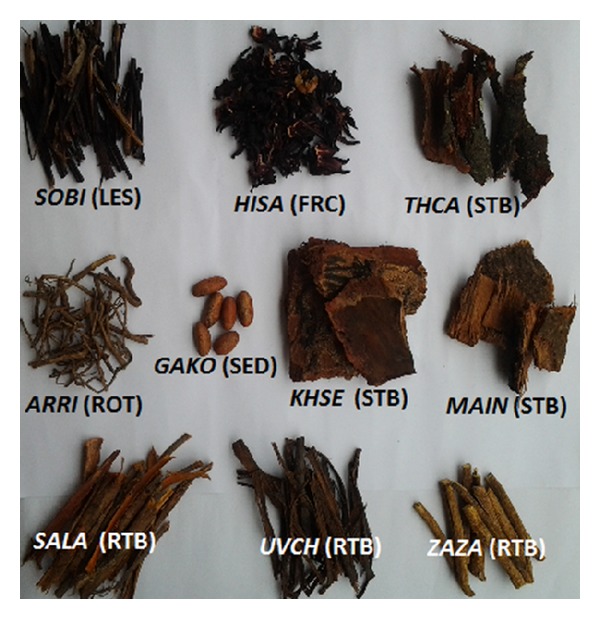
Samples of the herbs used in combination as haematinic herbal formulation (Haematol-B) in Ogbomoso, Nigeria. *SOBI*: *Sorghum bicolor*; *HISA*: *Hibiscus sabdariffa*; *THCA*: *Theobroma cacao*; *ARRI*: *Aristolochia ringens*; *GAKO*: *Garcinia kola*; *KHSE*: *Khaya senegalensis*; *MAIN*: *Mangifera indica*; *SALA*: *Sarcocephalus latifolius*; *UVCH*: *Uvaria chamae; ZAZA: Zanthoxylum zanthoxyloides*; LES: leaf sheath; FRC: fruit calyx; STB: stem bark; ROT: root; SED: seed; RTB: root bark.

**Figure 3:**
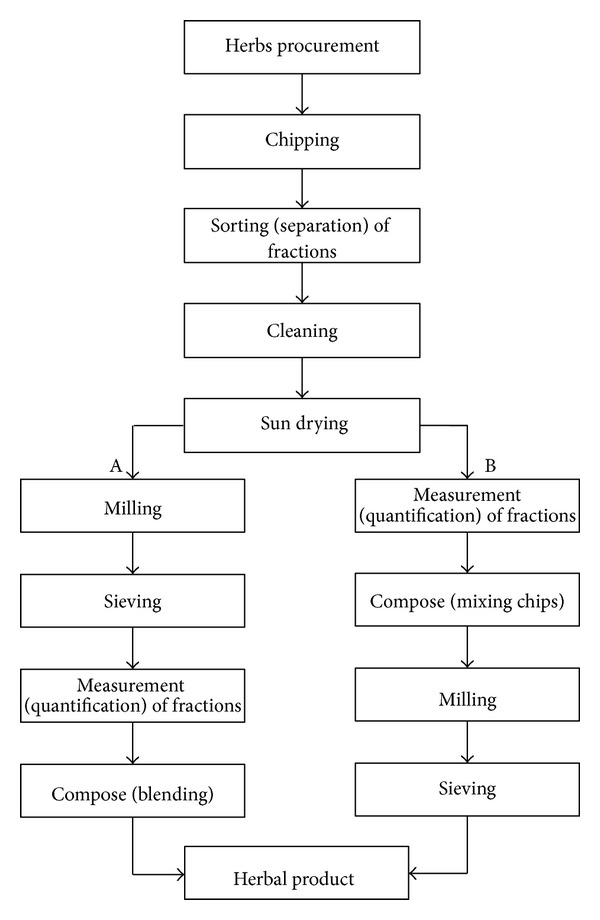
Flow chart of the processes for manufacturing powdered antimalarial and haematinic herbal remedies by the herbal practitioners in Ogbomoso, Nigeria. Option A was adopted by 84%; Option B was adopted by 16% of the 25 respondents.

The results of this study are shown in Figures 1, 2, and 3 and Tables 2 to 5. A wide variation in the botanical constituents and their relative quantities in the two herbal remedies was encountered during the survey. Between 1 and 14 different plant species were identified as constituents of AMHRs while those of HAHRs ranged between 1 and 12. Similar observations were made by Odugbemi et al. [20] with regard to malaria therapy in Okeigbo, Ondo State, southwest of Nigeria. The multistage preparation of the powdered remedies also followed diverse protocols, but with two main possible alternatives, namely, A and B. The majority (i.e., 84%) of the HPs usually adopted alternative A while alternative B was common with 16%, who incidentally were small scale manufacturers (Figure 3). The first alternative was probably more preferable because of the merit that the herbal fractions could more accurately be measured out in the desired proportions. Moreover, according to the respondents, milling of the fractions separately was advantageous because an herb so milled was usable in the formulation of more than one type of herbal remedies. The practice also ensured homogeneity of surface area in the final powdered product since two different herbal materials might not respond equally to the milling process. These observations are in consonance with the bottlenecks to polyherbal formulations identified by Mallavadhani [21], namely, large variations in different samples of the same formulation, variations in ingredients, variation in quantity of the ingredients, and lack of uniform manufacturing protocols.

The composition of antimalarial and haematinic herbal remedies from Ogbomoso in parts per hundred is shown in Tables 4 and 5, respectively. Not only have the botanicals making up these remedies been documented from this study, but also their relative quantities have been objectively defined. On this account, the two remedies were, respectively, named Maloff-HB and Haematol-B herbal formulations for the purpose of proper identification. On the question whether these formulations are representative of the practice in the study area, the answer is in the affirmative for two reasons. In the first place, the results were a product of wide consultations among referred individuals in the act of local herbal formulation and secondly, the formulations obtained from them have been statistically screened to an acceptable level of consistency (Tables 2 and 3).

As expected, the results of this study have shown the qualitative and quantitative variations in the two herbal formulations considered to be diverse. However, the most typical lists of herbs for AMHRs and HAHRs in this part of Nigeria have been statistically identified and quantitatively characterized (Tables 4 and 5). These efforts were not meant either to prejudice the practice in the study location or to force the manufacturers to conform to the most popular list of plants for each remedy. In fact one would recommend that all the variants of the two herbal formulations should be given adequate attention if the holistic view of the situation on ground is intended. But then, for any form of herbal drug standardization to be valid and workable, it should be based on the representative practice in the localities that offer the remedy.

Of the 49 herbal product outlets identified across the 41 healers initially interviewed, 28 and 21 offered AMHRs and HAHRs, respectively. Similarly, 19 and 14 were, respectively, identified across the 29 healers subsequently considered for analysis. This implies that the newly standardized Maloff-HB included the listed nine species of plants (Table 4) that were used by 68% of the outlets while the standardized Haematol-B included the 10 identified species that were favoured by 67% of the outlets. A total of nine (32%) antimalarial and seven (33%) haematinic herbal products outlets were at variance with the standardized formulations in terms of the composition of their recipe. While Maloff-HB and Haematol-B can be said to be representative of the practice in Ogbomoso in terms of the quality and quantity of their composition, the proportion of the dissenting practice would not allow the information therefrom to be ignored in future investigations.

Single herbs are the common form in which herbal formulations are presented and used. This practice appears to be the easiest way to consume herbs and most economical method of presenting them to individuals as well as herbal supplement manufacturers [22]. From all the herbalists contacted during the study, the names *Enantia chlorantha *and *Alstonia boonei* consistently occurred in the list of herbs for antimalarial remedies and so did *Sorghum bicolor* in the list for haematinic remedies. These findings are consistent with those of Odugbemi et al. [20] and could be a pointer to the general opinion that a single or at most two herbs could be potent enough to cure one or more ailments [23–25].

With great experience and knowledge of herbs spanning many centuries, indigenous people are believed to have learnt that some herbs required the inclusion of other ingredients (and also other herbs) as catalyst or else the brew of the herbs being used for therapeutic purposes was ineffective [26]. This discovery probably marked the origin of polyherbal or multiherbal formulations, which are obtained by subjecting herbal ingredients from a number of specified medicinal plant parts to various processes such as extraction, distillation, expression, fractionation, purification, concentration, and fermentation [21]. It is however important to note that, as we frequently see, many herbs go into a medicinal preparation, so we do have certain medicinal plants, each having multiple therapeutic uses [23, 27].

Mallavadhani [21] identified two key parameters of herbal drugs, namely, standardization and quality evaluation. While quality evaluation pertains to requirements for authenticity, purity, and safety, standardization deals, among other things, with assay, that is, analyzing herbal drug preparation and adjusting them to a defined content of a constituent. To that extent, herbal product standardization has been defined as the body of information and controls necessary to produce materials of reasonable consistency [28]. Rukangira [6] believes that the quality of traditional medicines is highly dependent on individual practitioner, who in his wisdom determines the correctness of identity and quantity of plant parts, and, as a result, there is no guarantee of the authenticity and quantity of plant material used in the preparation. To this extent, there is a need to select proper and appropriate technologies for the industrial production of medicines such that the effectiveness of the preparation can be ensured. One of the ways to achieve this goal is to minimize the inherent variation in natural product composition through quality assurance practice applied to manufacturing processes and techniques.

**Figure 4:**
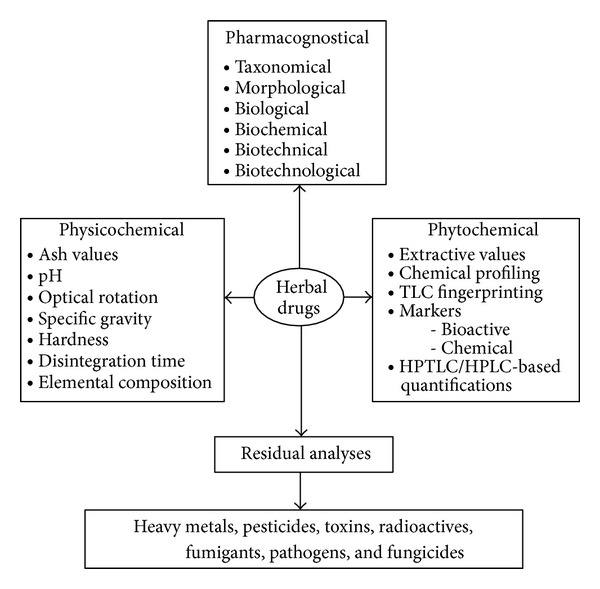
The triple P-based protocols along with residual analyses for standardization and quality control of herbal drugs (source: [21]).

Efforts of the World Health Organization and a number of countries dealing with herbal drugs have yielded the triple P-based protocols for standardization and quality control of herbal drugs, that is, pharmacognostical, physicochemical, and phytochemical, along with residual analyses (Figure 4). Since standardization of herbal medicines arose out of the need to create a uniform product for clinical trials [29], this present study can be said to have contributed a piece to the standardization of herbal medicine in Nigeria. It has qualitatively and quantitatively defined the botanical constituents of antimalarial and haematinic powdered herbal formulations commonly used in Ogbomoso, southwest of Nigeria.

## 4. Conclusion and Recommendation

Antimalarial (Maloff-HB) and haematinic (Haematol-B) powdered herbal formulations from Ogbomoso, Nigeria, have been botanically characterized and quantified. An aspect of the pharmacognostical approach to their standardization has thus been addressed. It would therefore be of great interest if researchers could work further to evaluate the pharmaceutical, phytochemical, and microbial qualities of Maloff-HB and Haematol-B with a view to considering them for general acceptability.

## Figures and Tables

**Table 1:** Local herbal product outlets visited for AMHRs and HAHRs in Ogbomoso, Nigeria.

	Local government area	Number of outlets		
		AMHRs	HAHRs	Total
1	Ogbomoso North	3	4	7
2	Ogbomoso South	3	1	4
3	Surulere	2	1	3
4	Oriire	2	3	5
5	Ogo-Oluwa	4	2	6

	Total	14	11	25

AMHRs: antimalarial herbal remedies; HAHRs: haematinic herbal remedies.

**Table 2:** Similarity matrix based on correlation coefficients of data collected from antimalarial herbal remedy outlets in Ogbomoso, Nigeria.

	OT1	OT2	OT3	OT4	OT5	OT6	OT7	OT8	OT9	OT10	OT11	OT12	OT13	OT14
OT1	1													
OT2	0.844**	1												
OT3	0.737*	0.978**	1											
OT4	0.806*	0.991**	0.978**	1										
OT5	0.733*	0.978**	1.00**	0.977**	1									
OT6	0.815*	0.984**	0.968**	0.991**	0.968**	1								
OT7	0.796*	0.993**	0.991**	0.992**	0.991**	0.986**	1							
OT8	0.676*	0.949**	0.959**	0.977**	0.959**	0.958**	0.962**	1						
OT9	0.764*	0.979**	0.995**	0.970**	0.944**	0.958**	0.988**	0.937**	1					
OT10	0.727*	0.979**	0.992**	0.987**	0.993**	0.976**	0.989**	0.979**	0.981**	1				
OT11	0.481^ns^	0.835**	0.886**	0.876**	0.884**	0.821**	0.958**	0.936**	0.866**	0.906**	1			
OT12	0.671*	0.948**	0.969**	0.875**	0.970**	0.970**	0.965**	0.987**	0.944**	0.987**	0.911**	1		
OT13	0.789*	0.992**	0.991**	0.986**	0.992**	0.980**	0.996**	0.958**	0.989**	0.985**	0.853**	0.958**	1	
OT14	0.923*	0.949**	0.890**	0.935**	0.886**	0.916**	0.992**	0.872**	0.899**	0.883**	0.743**	0.842**	0.926**	1

**Correlation is significant at the 0.01 level (2-tail); *significant at the 0.05 level (2-tail); ns: not significant; OT1, OT2, OT3,…, OT14: antimalarial herbal remedy outlets 1 to 14 used for the study.

**Table 3:** Similarity matrix based on correlation coefficients of data collected from haematinic herbal remedy outlets in Ogbomoso, Nigeria.

	OT1	OT2	OT3	OT4	OT5	OT6	OT7	OT8	OT9	OT10	OT11
OT1	1										
OT2	0.981**	1									
OT3	0.971**	0.985**	1								
OT4	0.987**	0.964**	0.964**	1							
OT5	0.966**	0.979**	0.999**	0.959**	1						
OT6	0.978**	0.988**	0.997**	0.975**	0.996**	1					
OT7	0.975**	0.951**	0.957**	0.991**	0.951**	0.972**	1				
OT8	0.961**	0.958**	0.935**	0.952**	0.931**	0.935**	0.911**	1			
OT9	0.953**	0.949**	0.984**	0.954**	0.986**	0.981**	0.954**	0.916**	1		
OT10	0.985**	0.977**	0.982**	0.979**	0.977**	0.988**	0.981**	0.917**	0.963**	1	
OT11	0.951**	0.980**	0.991**	0.941**	0.988**	0.989**	0.946**	0.895**	0.965**	0.977**	1

**Correlation is significant at the 0.01 level (2-tail); OT1, OT2, OT3,…, OT11: haematinic herbal remedy outlets 1 to 11 used for the study.

**Table 4:** Botanical characterization and % composition (wt/wt) of antimalarial herbal formulation (Maloff-HB) from Ogbomoso, Nigeria.

	Plant species	Family	Indigenous name (Yoruba)	Part used	Total weight (g)	Mean weight (g)	Parts per 100
1	*Enantia chlorantha * Oliv.	Annonaceae	Awopa/Dokita igbo	Stem bark	19248.6	1374.9 (1039.5–2005.5)	30
2	*Alstonia boonei* De Wild	Apocynaceae	Ahun	Stem bark	12832.3	919.5 (527.5–1232.0)	20
3	*Calliandra haematocephala* Hassk	Fabaceae	Tude	Root	6430.6	459.8 (310.0–740.0)	10
4	*Mangifera indica* L.	Anacardiaceae	Mongoro	Stem bark	3077.2	460.5 (137.8–380.0)	10
5	*Okoubaka aubrevillei* Phelleg & Nomand	Santalaceae	Igi nla	Stem bark	5092.9	366.3 (134.3–523.3)	8
6	*Sarcocephalus latifolius* (J. E. Smith) E. A. Bruce	Rubiaceae	Egbesi	Root bark	5140.2	367.2 (232.0–554.7)	8
7	*Parquetina nigrescens* (Afz.) Bullock	Periplocaceae	Ogbo	Root bark	3820.5	273.9 (195.0–357.5)	6
8	*Cassytha filiformis *L.	Lauraceae	Omonigelegele	Vine	2039.1	181.7 (119.9–278.0)	4
9	*Pterocarpus osun* Craib	Papilionaceae	Igi osun	Stem bark	2686.0	185.7 (127.8–286.8)	4

	Total					4589.5	100

wt/wt: weight-for-weight; *n* (number of outlets) = 14; values in parentheses are the ranges of the measurements.

**Table 5:** Botanical characterization and % composition (wt/wt) of haematinic herbal formulation (Haematol-B) from Ogbomoso, Nigeria.

	Plant species	Family	Indigenous name (Yoruba)	Part used	Total weight (g)	Mean weight (g)	Parts per 100
1	*Sorghum bicolor* Moench	Poaceae	Oka baba	Leaf sheath	13371.1	1215.4 (917.0–1632.0)	30
2	*Hibiscus sabdariffa* L. (red variety)	Malvaceae	Isapa pupa	Fruit calyx	9104.0	827.6 (460.0–972.0)	20
3	*Theobroma cacao* L.	Sterculiaceae	Koko	Stem bark	4565.2	415.0 (241.5–494.5)	10
4	*Aristolochia ringens* Vahl.	Aristolochiaceae	Akogun	Roots	3174.2	288.6 (140.4–453.0)	7.0
5	*Garcinia kola* Heckel	Guttiferae	Orogbo	Seed	2473.4	225.0 (104.0–309.5)	5.5
6	*Khaya senegalensis* (Desr.) A. Juss.	Meliaceae	Agano	Stem bark	2505.0	227.7 (211.0–283.0)	5.5
7	*Mangifera indica* L.	Anacardiaceae	Mongoro	Stem bark	2519.2	229.0 (176.0–282.0)	5.5
8	*Sarcocephalus latifolius * (J. E. Smith) E. A. Bruce	Rubiaceae	Egbesi	Root bark	2508.9	228.3 (97.0–325.0)	5.5
9	*Uvaria chamae * P. Beauv.	Annonaceae	Eruju	Root bark	2491.9	227.5 (173.0–340.3)	5.5
10	*Zanthoxylum zanthoxyloides* (Lam.) Zepern & Timler	Rutaceae	Ata	Root bark	2480.3	225.7 (120.4–303.7)	5.5

	Total					4109.8	100

wt/wt: weight-for-weight; *n* (number of outlets) = 11; values in parentheses are the ranges of the measurements.
